# The match between institutional elderly care management research and management challenges - a systematic literature review

**DOI:** 10.1186/1478-4505-10-35

**Published:** 2012-11-08

**Authors:** Kaija Kokkonen, Sari Rissanen, Anneli Hujala

**Affiliations:** 1 Department of Health and Social Management, University of Eastern Finland, Kuopio Campus, P.O. BOX 1627, Kuopio, FI, 70211, Finland; 2 Department of Social Sciences, University of Eastern Finland, Kuopio, Finland

**Keywords:** Management, Leadership, Policy, Institutional care, Long-term care, Nursing home, Elderly care, Aged care, Health services for aged, Geriatric nursing

## Abstract

**Background:**

Elderly care practice and its management together with policy and research play a crucial role in responding to increasing challenges in institutional care for elderly people. Successful dialogue between these is necessary. The purpose of this systematic literature review is to compare how institutional elderly care management research meets the care challenges currently emphasized in international long-term care policy documents.

**Methods:**

This paper was based on a systematic literature review. After screening 1971 abstracts using inclusion/exclusion criteria, 58 refereed articles published between 2000 and 2010 remained for analysis. The articles were analyzed using theory-based content analysis by comparing the results to the framework based on analysis of international long-term care management policy documents.

**Results:**

The current challenges of long-term care management identified from policy documents were Integrated Care Management, Productivity Management, Quality Management, Workforce Management and ICT Management. The research on institutional elderly care management responded somewhat to the challenges mentioned in policy documents. However, some of the challenges were studied broadly and some were paid only minor attention. Further, only few studies focused on the core items of challenges addressed in policy documents.

**Conclusions:**

Institutional care management research needs to focus more on challenges in integrated care, productivity, ICT and division of labor. Managers, researchers and policy-makers should assume more active collaborative roles in processes of research, policymaking and policy implementation. In addition managers’ and policymakers’ scientific literacy needs to be enhanced.

## Background

The purpose of the review is to compare institutional elderly care management research to the care challenges currently emphasized in international long-term care policy documents. Thus this review concentrates on the dialogue between policy, management, research and practice in the field of institutional elderly care. The task of global-level long-term care policy is to provide objectives for management and practices. Institutional care management from its part seeks to implement these objectives in practice. The role of research is to evaluate policy objectives, management and practices. Finally practice together with management and research should provide policymakers with feedback information. Consequently all these issues are inextricably linked.

The following reasons can be evinced as the basis for this review article: Firstly it is commonly accepted that long-term care for elderly people has faced many challenges globally. Population ageing also increases the number of dependent elderly people in need of institutional care, which needs increasing financial resources. In addition, common attitudes toward the quality of long-term care are changing in a more demanding and client-oriented direction. The key challenge for governments internationally is to find a way to bridge the gap between expectations for better quality of care and resources made available for delivering care [1].

Secondly, public long-term care policies nationally and internationally steer the production of services for different groups of clients. An increasingly complex world of interrelated problems needs to strengthen the link between evidence, policy and practice, also in the field of public policy [2,3]. Despite the requirement for evidence-based policy decisions, the implementation of evidence-based policy in practice is complicated. As noted by Davis and Howden-Chapman [4], we have convincing evidence of policy in the making and implementation, but little indication of the cumulative research that might have inspired and guided these developments. In the case of medical practice there is a contrary example: a considerable amount of research evidence on the quality of practice, but little sign of its widespread implementation. This article is also an attempt to guide and increase the cumulative knowledge of results of management research to support both long-term care policy and management in institutional care practice.

Thirdly, management research in institutional elderly care offers scientific information about management in this specific context. It has been concluded that a field of management science should develop and identify more useful applications for the practice of managing [5,6] In addition David et al. [4] mentioned researchers’ responsibility to see their research translated into policy. This supports so-called evidence-based management, which has been an important part of the current debate about management in nursing and social sciences.

This article presents the results of a systematic literature review of institutional elderly care management research. The purpose of the review is to compare how institutional care management research matches the long-term care challenges currently emphasized in international long-term care policy for elderly people. Defining the concepts of management, long-term care and institutional care is complex, from the perspectives of both research and practice see [6,7]. In this review, management is seen from a manager-centered perspective, in which organizational level management is in the focus. Long-term care refers to formal care of elderly people needing support in many functions for a prolonged period of time, and includes care both in home or institutional settings. Institutional care refers to care which is given in institutions such as nursing homes. So, institutional care is only a part, but often the most resource intensive, of long-term care and was thus chosen for the focus of the literature review. However, because international policy documents concerning only institutional elderly care were not available, the policy documents were searched for in the field of the broader concept of long-term care. In addition, institutional care as the context of systematic literature review brought about a more solid search of data to review than a broader approach.

The structure of this article is as follows: In the following section the international policy documents chosen for this study and the long-term care challenges based on these documents are presented. The Methods section describes process of the systematic literature review and basic information of the articles in this literature review. The section on Results and Discussion illustrates the match between institutional care management research and current long-term care challenges. Finally the section entitled Conclusions includes recommendations for institutional care management research and its usability in long-term care for elderly people.

### Conceptual framework

National and global actors have produced different kinds of policy documents on long-term care for the elderly, including institutional care. The first criterion for selecting international long-term care documents for this review was their timeframe, less than six years old. The second and third criteria were that the documents should include recommendations for future actions based on research and the broad consensus of a multi-professional team. On this basis five European or international level policy documents were chosen for inclusion in this review as a framework presenting the present challenges in long-term care for elderly people. These documents represent three major international organizations: the EU, the WHO and the OECD, one of whose tasks is to produce information and make recommendations for future actions for the national policies for health and social care systems. Next these five policy documents are briefly presented:

a) *The Policy Framework for Integrated Care for Older People*[8] offers recommendations for improving the integration of care for older people, especially at the national, regional, organizational and individual level. The document is developed by consensus in the Carmen network (Care and Management of Services for Older People in Europe Network). This thematic network has been managed by the European Health Management Association (EHMA) and has been funded by the European Commission under the RTD program ‘Quality of Life and Management of Living Resources 1998–2002’. see also [9-12].

b) *LongTerm Care in the European Union*[13] includes an analysis of the main challenges in long-term care in different European countries and strategies to ensure the overall political commitment to meet the individual needs of elderly people in long-term care practice. The document is based on the experiences of national authorities of long-term care and is funded by EU.

c) *Silver paper*[14] provides recommendations for future actions in basic biological research on ageing and in health promotion and clinical care for elderly people. The document is a consensus report of the outcome of the European Summit on Age Related Disease, held under the European Union. It is based on a consensus of European politicians, geriatrists, gerontologists and the health ministries of Member States.

d) *How can health systems respond to population ageing*? [15] offers research evidence for policymakers and health system managers to develop health systems to respond to population ageing. This document is part of the new policy series of the Health Evidence Network (HEN) of the WHO and the European Observatory on Health Systems and Policies.

e) *Help Wanted*? *Providing and Paying for LongTerm Care*[16] focuses on the implications of ageing population for labor markets and the financing of long-term care services. The policy document calls for a comprehensive approach to long-term care and is based on an OECD report on the analysis of economic development and health and well-being of elderly people.

### Current challenges to long-term care emphasized in policy documents

The focal challenges of long-term care identified from the policy documents are presented in Table 1. These challenges were those most strongly addressed in all documents and identified by the researchers through content analysis of the policy documents. The analysis was done by searching for challenges to long-term care, which in this review are considered from the perspective of management. The challenges were grouped into five categories: Integrated Care Management, Productivity Management, Quality Management, Workforce Management and ICT Management. In the following, these five main categories and their sub-categories are briefly presented.

**Table 1:** Challenges of long-term care management emphasized in international policy documents

**Management challenges**	**Policy framework for integrated care for older people: 2004 (EHMA) a**	**Long-term care in the European Union: 2008 (EU) b**	**Silver paper. 2008 (EU) c**	**How can health systems respond to population ageing? 2009 (WHO) d**	**Help wanted? providing and paying for long-term care: 2011(OECD) e**
**Integrated Care**	“Services need to be delivered across organisational boundaries, with clear access points and pathways” p.5	“More attention to introducing measures that will make different services work more effectively together” p.12	“Multidisciplinary care, adapted to the needs of each individual, their families and caregivers” p.55	“Provide support to relatives, enabling them to continue to provide help and support for elderly people” p.20	“Addressing the growing need for long-term care requires a comprehensive vision” p.1
· within organization					
· between organizations					
· with informal care					
**Productivity Management**	“Improving service system efficiency for service users with complex needs by taking a ‘whole-system’ approach” p.3	“ A preventive approach, integration and use of ICT help to keep costs under control” p.10	“High quality services…and make them financially sustainable” p.55	“Mitigate the potential cost pressures related to long-term care” p.14	“With rising costs, seeking better value for money in long-term care must be a priority” p.7
· financial sustainability					
· efficiency of care					
**Quality Management**	“Maximising older people’s quality of life, independence and control” p.3	“A focus on more comprehensive quality assurance involving issues such as patients’ rights” p.7	“A quality of care assurance system should be implemented” p.55		“Higher expectations that the final few years of life must have” p.1
· quality assurance system					
· setting the goals					
· assessment the quality					
**Workforce Management**	“Workforce development to train people in new approaches – for example, working in networks and partnerships” p.31	“Attracting the right personnel, especially given the medical and social care expertise required” p.15	“Strengthening capacity building in training of professionals working with older people“ p.54		“Higher turnover . endanger both access and quality of services.” p.5 “Delegation of tasks” p.5
· training					
· stability and accessibility					
· division of labor					
**ICT Management**	“Developing effective shared IT and information systems as part of the infrastructure to integrated care?” p.31	“Effective promotion of care.requires the efficient use of ICT”. p.11	“Technological aids have to be gradually incorporated in all aspects of care” p.55		“The use of ICT to reduce indirect workload” p.5
· technology aid					
· care process tools					
· management tools					

Firstly, the need to improve integrated care in long-term care was the most strongly and frequently emphasized challenge in all five documents. In recent decades much of the end-of-life care for elderly people has been shifted from hospitals to long-term care institutions and this trend is likely to continue. This demands closer integration of the long-term care sector between health and social professionals and organizations within the organization, as well as across different levels of care organizations (documents a, b, c, d, and e). Political documents also increasingly stressed the importance of integrating informal and formal care (documents a, c, e) and better coordination of national, regional and local authorities and services (document e). Integrated care was seen as a crucial issue to ensure the quality of care and a client-centered approach (documents a, b, d, and e) and to improve the effectiveness of care (documents a, and c).

Secondly, improving productivity of long-term care was mentioned in all five documents. The documents stressed that ageing is expected to bring about an increase in public spending on long-term care (documents d, and e). Improving and assessing the productivity of long-term care were deemed essential to ensure its sustainability (documents a, b, and d) and the improvement of the quality of care (document a). The division of labor, ICT, self-care management services (document e), integrated care (document a) and effective health interventions (document c) and adequate systems of long-term care (document d) were mentioned as tools for more productive long-term care.

Thirdly, the demand for better quality of long-term care emerged in four policy documents (documents a, b, c, and e). The most common references were concern regarding poor quality of long-term care, which also tends to vary widely between and within countries (documents b, and e). Good quality care was deemed essential for frail elderly people but also as a means of increasing staff’s intent to stay (document e). The main challenges for quality improvement in long-term care mentioned in the documents were need for permanent quality assurance systems with shared visions and clear goals (documents a, b, and e), need for valid quality indicators (documents a, and e) and proper assessment tools (documents a, and b). Measurement of quality should develop from compliance with certain minimum requirements towards a focus on more comprehensive quality assurance involving issues such as patients’ rights, equality and abuse of elderly people (document b). In addition in the Silver Paper (document c) the need for evidence based practices in modern clinical gerontology and geriatric medicine was articulated.

Fourthly, the workforce challenges were noted in four policy documents (documents a, b, c, and e). The most mentioned workforce challenge was education and training (documents a, b, and c). Continuing training was claimed to promote quality improvement in long-term care (documents b, and c), workforce stability (document b), and implementation of new methods of work like integrated care (document a). The second challenge mentioned regarding the workforce was growing shortages of medical, nursing and social care labor (documents b, and e). Training (document b), better working conditions and better pay (documents b, and e) were mentioned as possible means to relieve workforce shortages. Additionally, division of labor by reorganizing tasks, leaving simpler tasks to less qualified workers or expanding care provision roles were considered as possibilities to alleviate the need of workforce (document e).

Fifthly, the challenge of exploiting information and communication technology (ICT) tools was mentioned in four policy documents (documents a, b, c, and e). The progress of the introduction of ICT has been slow in long-term care and the pressure to develop and incorporate the use of ICT tools is obvious (documents a, b, c, and e). The policy documents named three dimensions in the use of ICT tools: 1) tools which improve the quality of the private lives of elderly people e.g. technology aids (documents b, and c), 2) tools which enhance care processes, like telemedicine and health records (document e) and 3) tools which support long-term care management e.g. tools for evaluation and planning (document a). Better quality of care (documents a, and c) and cost savings (documents b, and e) were mentioned among the advantages of ICT in long-term care.

To conclude, the most obvious long-term care management challenges derived from policy documents were: 1) integrated care management 2) productivity management, 3) quality management, 4) workforce management and 5) ICT management. These will be used as an analytical framework through which to contemplate management research.

## Methods

### The systematic literature review

The articles on institutional elderly care management were analyzed using the process of systematic literature review. The purpose of the systematic literature review is to produce evidence-based and cumulative information using a special and transparent review protocol [3]. This review follows Petticrew’s and Roberts’ [17] p.284-287 review protocol, which includes clear review questions, a well-defined search strategy, definitions of inclusion and exclusion criteria, completing data extraction sheets for each study and a synthesis of primary studies. The following methodological steps were taken in the linear process.

The search for articles was conducted in databases from two electronic sources, namely Web of Science and EBSCO (Cinahl, SocIndex, Business Source Elite, AcsPre). The search was conducted together with a library information specialist. The search words used (and word combinations) were “aged care” OR “elder*care” OR “nursing home” OR “long-term care” OR “institutional care” OR “health services for the aged” (Mesh) OR “geriatric nursing” AND “elder*” OR “aged” OR “dement*” OR “old*people*” AND “management” OR “leadership” OR “manager” OR “leader” OR “operational manage*” OR “middle manage*” OR “strategic manage*” OR “front-line manage*”. The searches yielded 1971 relevant citations.

The main inclusion criterion was the relevance of the paper to the purpose of the review. This review of articles focused on management from the perspective of managers and their actions. This means, for instance, the actions of charge nurses, nursing directors, nurse managers, supervisors and managers. Only articles that addressed the empirical study of institutional care management were included. Other inclusion criteria were that a paper was published in English and it was a refereed article. Both qualitative and quantitative studies were included, but review articles were not included in this review. Articles published from 2000 to November 2010 were searched for.

In the second step 1971 abstracts were read and reread and 1841 of these abstracts, namely duplicates, non-refereed articles, language other than English or unrelated aim of study, such as illness management and instrument assessment were eliminated. In the third step 130 potentially relevant studies were screened on the basis of full-text. In the fourth step 72 full-text articles were excluded due to topics of study not relevant to the purpose of the review. Fifty-eight refereed articles remained for analysis.

The following background information on the articles was extracted: authors, year of publications, journal, country where the study was conducted and method of data collection and analysis. In addition, the theoretical backgrounds of the studies were categorized. The findings of the studies were then analyzed using theory-based content analysis by comparing the results to the framework mentioned above. Suitable studies were classified under the five categories named in the framework. On the next level studies in one framework category were divided into sub-categories of the framework according to the main result. After that those studies which did not fit into these categories where categorized into two other separate data-based categories which will be presented at the end of the Results and Discussion section. A few results of the studies were connected to more than one category, for example quality work management and ICT management, in which case the main category was chosen according to the main emphasis of the article. A categorization of the studies in the literature review is presented in Table 2.

**Table 2:** Categorization of studies in the systematic literature review

**Authors**	**Journal**	**The main result**	**Categories of management challenges**	**Sub-categories of management challenges**
Henriksen E, Rosenqvist U, 2003 [18]	Health and Social Care in the Community	The different ways of understanding elderly care services showed a complex and fragmented in organization lacking clear goals, structures and leadership.	Integrated Care Management	Co-operation between organizations
Henriksen E, Selander G, Rosenqvist U, 2003 [19]	Health Policy	All participants agreed on four key visions for the healthcare of the elderly: see the person, see the individual resources, see the encounter and see yourself. Other findings indicated that (a) care of older persons was governed by diverse interests, (b) the organization lacked clear leadership and comprehensive goals, (c) the organization was fragmented and (d) the lack of skilled staff members to meet patient needs.	Integrated Care Management	Co-operation between organizations
Miller SC, 2010 [20]	Journal of Palliative Medicine	Nursing home-hospice collaborators were philosophically and otherwise aligned; they had similar missions, understood their differing approaches to care, and administrators demonstrated an openness and support for the collaboration.	Integrated Care Management	Co-operation between organizations
Wilson CB, 2009 [21]	Health and Social Care in the Community	The key factors influencing relationships that emerged were leadership, continuity of staff, personal philosophy of staff and contribution of residents and families.	Integrated Care Management	Co-operation with informal care
Madas E, North N, 2000 [22]	Australian Health Review	The most prominent issues facing managers were considered to be inadequate funding to match the growing costs of providing long-term care and occupancy levels.	Productivity Management	Financial sustainability
Rantz MJ, Hicks L, Grando V, et.al. 2004 [23]	The Gerontologist	For nursing home to achieve good resident outcomes, they must have leadership that is willing to embrace quality improvement and group process and see that basics of care delivery are done for residents. Good quality care may not cost more than poor quality care.	Productivity Management	Efficiency of care
Kjos BO, Botten G, Gjevjon ER, Romoren TI, 2010 [24]	International Journal for Quality in Health Care	Quality work was fragmented rather than comprehensive and systematic.	Quality Management	Quality assurance system
Baier R, Butterfield K, Patry G, et.al. 2009 [25]	Journal of American Geriatrics Society	Nursing homes with ambitious targets demonstrate greater improvement than their peers selecting less-ambitious targets.	Quality Management	Setting the goals
Parmelee PA, Bowen, SE, Ross, A, Brown H, Huff J, 2009 [26]	Journal of the American Medical Directors Association	Although quantitative ratings were generally positive, qualitative analysis yielded a number of emergent themes regarding data accuracy, team functioning, timeliness of assessments, and validity of MDS tool itself.	Quality Management	Assessment the quality
Zimmerman S, Gruber-Baldini AL, Hebel JR et.al. 2002 [27]	Journal of American Geriatrics Society	High rates of hospitalization for infection were associated with for-profit ownership, chain affiliation, poor environmental quality, lack of resident privacy, lack of administrative emphasis on staff satisfaction, and low family/friend visitation rates.	Quality Management	Leadership and resident outcomes
Anderson RA, Issel LM, McDaniel RJ, 2003 [28]	Nursing Research	Each management practice explained one or more of the resident outcomes.	Quality Management	Leadership and resident outcomes
Rantz MJ, Grando V, Conn V, et.al. 2003 [29]	Journal of Gerontological Nursing	Directors of nursing in facilities with good outcomes are much more likely to have been in their jobs for many years. Facilities with good outcomes are more likely to use group processes for decision-making and most of these facilities have active quality improvement programs.	Quality Management	Leadership and resident outcomes
Neily J, Howard K, Quigley P, Mills PD, 2005 [30]	Joint Commission Journal on Quality and Patient Safety	Leadership support, experience with quality improvement and teamwork skills, and skills gained from the project were correlated with team s’ abilities to achieve and maintain success.	Quality Management	Leadership and resident outcomes
Scott-Cawiezell J, Main DS, Vojir CP, et.al. 2005 [31]	Health Care Management REVIEW	The staff in the high-scoring nursing homes felt valued for their contribution to the provision of high quality care. Contrary to this perspective, leadership from low-scoring nursing homes did not emphasize the value of staff and discussed them as their greatest concern.	Quality Management	Leadership and resident outcomes
Forbes-Thompson S, Leiker T, Bleich MR, 2007 [32]	Health Care Management REVIEW	Leaders in higher-performing homes behaved congruently with the nursing home’s stated and lived mission by fostering connectivity among staff, ample information flow, and the use of cognitive diversity.	Quality Management	Leadership and resident outcomes
Dellefield ME 2008 [33]	Journal of Nursing Care Quality	Supervisors and managers who understood the work of caregiving, respected and valued it, and communicated this to subordinates were likely to be described as providing effective supervision and management.	Quality Management	Leadership and resident outcomes
Gnaedinger N, 2003 [34]	Journal of Social Work in Long-Term Care	Keys to successfully implementing resident-centred approach were identified as: higher staff-to-resident ratios, effective leadership, formal involvement of front line staff in decision making, on-going education and training for all provides, and some rotation of staff scheduling.	Quality Management	Leadership and successful implementation
Jeong SY-S, Keatinge D 2004 [35]	Journal of Nursing Management	The impact of policy change on nursing staff and their practice depended on the management’s leadership in interpreted the new policy and implemented innovative strategies in order to meet its requirements.	Quality Management	Leadership and successful implementation
Lee RH, Wendling L 2004 [36]	American Journal of Medical Quality	High leadership turnover and limited leadership training make it difficult for nursing homes to sustain effective Quality Improvement programs.	Quality Management	Leadership and successful implementation
Morgan DG, Stewart NJ, D'Arcy C, Cammer AL 2005 [37]	Canadian Journal of Nursing Leadership	The key finding was the critical role of nursing leadership and supervision in creating and sustaining the unit. Four key leadership activities were identified: perpetual reinforcement, enforcement of SCU goals and ideals; support, guidance and mentoring of staff; empowerment of staff; and liaison/public relations.	Quality Management	Leadership and successful implementation
Scott-Cawiezell J, Vogelsmeier A, McKenney C, et.al. 2006 [38]	Nursing Forum	Nurse leaders can create an environment in which every member of the team feels a responsibility and an ability to ensure that residents are safe by improving communication and participation in decision making.	Quality Management	Leadership and successful implementation
Bostrom A-M, Wallin L, Nordstrom G, 2007 [39]	Journal of Evaluation in Clinical Practice	Four factors were significantly related to research utilization, namely: attitudes toward research, seeking research that is related to clinical practice, support from unit manager and access to research findings at work place.	Quality Management	Leadership and successful implementation
Ploeg J, Davies B, Edwards N, et.al. 2007 [40]	Worldviews of Evidence-Based Nursing	Staff, CRNs and administrators identified support from nurse managers and administrators at all levels of the organization as a key facilitator for successful guideline implementation.	Quality Management	Leadership and successful implementation
Cruttenden KE, 2006 [41]	Canadian Journal on Aging	Leadership strengths defined the roles for categories of staff and supported the capacity of each category to identify their learning needs.	Workforce Management	Training
Morgan JC, Konrad TR, 2008 [42]	The Gerontologist	Managers’, supervisors’, and participating NA’s consistent perceptions of improved quality of care and job quality, along with a promise of increased retention, suggest that interventions like WIN A STEP UP are beneficial.	Workforce Management	Training
Häggström E, Bruhn Å, 2009 [43]	Nurse Education Today	The management did not create conditions that made it possible to participate during working hours [education ].	Workforce Management	Training
Mitchell CM, Zimmerman S, Beeber AS, 2010 [44]	Alzheimer’s Care Today	The odds of attending the training increased with staff age, and the odds were higher for staff reporting more effective leadership and who worked in facilities that were more tolerant of residents who displayed behavioral symptoms.	Workforce Management	Training
Anderson RA, Corazzini KN , McDaniel RR, 2004 [45]	The Gerontologist	In nursing home with reward-based administrative climates, higher levels of communication openness and accuracy explained lower turnover of licensed vocational nurses and certified nurse assistants relative to nursing homes with an ambiguous climate.	Workforce Management	Staff stability
Hsieh P, Su H, 2007 [46]	International Journal of Nursing Studies	Major reason for staying were personal interest in caring elderly, good financial benefits from the facility and supportive leadership.	Workforce Management	Staff stability
Kemper P, Brannon D, Barry T, Stott A, Heier B 2008 [47]	The Gerontologist	Factors that affected project implementation included having demonstration resources; strong, stable leadership; strong coalition that included key stakeholders; neutral lead agency; clear goals; effective process; and a favorable state history and context.	Workforce Management	Staff accessibility
Donoghue C, Castle NG, 2009 [48]	The Gerontologist	NHAs who are consensus managers (leaders who are solicit, and act upon, the most input from their staff) are associated with the lowest turnover levels, 7% for RNs, 3% for LPNs, and 44% for NAs.	Workforce Management	Staff stability
Tourangeau A, Cranley L, Spence Laschinger HK, Pachis J 2010 [49]	Journal of Nursing Management	No relationship was found between leadership practices and job satisfaction or turnover intention.	Workforce Management	Staff stability
Wilson AA, 2005 [50]	Nursing Administration Quarterly	The management education program significantly increased their intent to stay in their current positions.	Workforce Management	Managers stability
Forbes-Thompson S, Gajewski B, Scott-Cawiezell J, Dunton N 2006 [51]	Western Journal of Nursing Research	Turnover of the NHA and DON is significantly and negatively associated with communication and teamwork.	Workforce Management	Managers stability
Holecek T, Dellmann-Jenkins M, Curry D, 2010 [52]	Journal of Applied Gerontology	An overall positive perception of the survey process was a significant predictor of administrator job satisfaction and job seeking.	Workforce Management	Managers stability
Kash BA, Naufal GS, Dagher RK, Johnson CE, 2010 [53]	Health Care Management REVIEW	Higher perceived salary competitiveness and level of empowerment were associated with reduced odds of intending to leave. Higher educational levels were associated with higher odds of intentions to leave.	Workforce Management	Managers stability
Siegel EO, Young HM, Mitchell PH, Shannon SE, 2008 [54]	The Gerontologist	Findings revealed (a) considerable variation in organizational resources, systems, and processes to support organization and operationalization of the supervisory role; and (b) limited evidence of nurses’ estimation of the potential benefits of training and organizational systems to support supervisory practice and the complexity of the supervisory role.	Workforce Management	Division of labor
Corazzini KN, Anderson RA, Rapp CG, et.al. 2010 [55]	Online Journal of Issues in Nursing	All of nurses who participated in the interviews could articulate benefits of delegation. Delegation was seen as solution to the fact that RN in leadership role could not do everything…delegated had a sense of empowerment by staff.	Workforce Management	Division of labor
Kjos BO, Botten G, Romoren TI, 2008 [56]	International Journal for Quality in Health Care	The technical component that requires training in tools and techniques was low. Results show …greater progress in implementing the general quality improvements components than they did in implementing the technical quality improvement components that require training in tools and techniques.	ICT Management	ICT management tool
**Authors**	**Journal**	**The main result**	**Data-Based Categories**	
Aroian JF, Patsdaugther CA, Wyszynski ME, 2000 [57]	Nursing Economics	DONs in LTCNF reported that they were most involved in roles/responsibilities related to nursing/heath services management and least involved in professional nursing and long-term care management	Leader’s own opinion of her/his skills, role, style	
Scott-Cawiezell J, Jones K, Moore L, 2005 [58]	Journal of Nursing Care Quality	Leaders were more often reported to reflect hierarchy value orientation, emphasizing efficiency of operations and following rules and procedures.	Leader’s own opinion of her/his skills, role, style	
Johansson G, Pörn I, Theorell T, Gustafsson B, 2007 [59]	Journal of Clinical Nursing	The first-line manager had three goals in her goal-profile in the following order of priority: (i) a nurse goal that she had strongly accepted and in which she had excellent control, (ii) an administrator goal that she had accepted and in which she had control, (iii) a leadership goal that she had not accepted and in which she did not have control.	Leader’s own opinion of her/his skills, role, style	
Shanley C, 2007 [60]	Journal of Organizational Change Management	The management of change is in the background of management thinking and practice in the industry [residential aged care].	Leader’s own opinion of her/his skills, role, style	
McGilton KS, Bowers B, McKenzie-Green B, et.al. 2009 [61]	Journal of Applied Gerontology	Themes that captures the following dimensions of the supervisory role in LTC include a) against all odds, getting through the day; (b) stepping in work; and c) leading and supporting unregulated care workers.	Leader’s own opinion of her/his skills, role, style	
Vesterinen S, Isola A, Paasivaara L, 2009 [62]	Journal of Nursing Management	Five categories of leadership style were discerned: visionary, coaching, affiliate, democratic, commanding.	Leader’s own opinion of her/his skills, role, style	
Nielsen K, Cleal B, 2010 [63]	Journal of Occupational Health Psychology	Line managers in elderly care experienced flow more often than accountancy line managers, and activities such as planning, problem solving, and evaluation predicted transient flow states.	Leader’s own opinion of her/his skills, role, style	
Abdelrazek F, Skytt B, Aly M, 2010 [64]	Journal of Nursing Management	FLMs’ perceptions of their leadership and management skills and psychological empowerment were quite high, whereas scores for job satisfaction and psychosomatic health were lower. FLMs had higher values in several factors variables in Egypt compared with in Sweden.	Leader’s own opinion of her/his skills, role, style	
Albinsson L, Strang P, 2002 [65]	Health and Social Care in the Community	The nearly unanimous opinions of the interviewees indicated that there was a great lack of daily leadership in the units.	Supportive and transformational leadership	
Scott-Cawiezell J, Schenkman M, Moore L, 2004 [66]	Journal of Nursing Care Quality	In terms of leadership, licensed practical nurses perceived less clarity of expectations, encouragement of initiative, and support than other groups.	Supportive and transformational leadership	
Hall L, McGilton KS, Krejci J, et.al. 2005 [67]	Journal of Nursing Administration	Many of behaviors identified as supportive by staff and supervisors in long-term care settings in this study can be linked to transformational leadership.	Supportive and transformational leadership	
McGilton KS, McGillis Hall L, Boscart V, Brown M, 2007 [68]	Canadian Journal of Nursing Leadeship	Forty-five percent of the total variance in job satisfaction of nurse supervisors was explained by supervisory support, stress and job category (registered nurse vs. registered practical nurse). Greater supervisory support was also associated with reduced job stress.	Supportive and transformational leadership	
Nielsen K, Randall R, Yarker J, Brenner S-O, 2008 [69]	Work & Stress	The results indicated that followers’ perceptions of their work characteristics did mediate the relationship between transformational leadership style and psychological well-being.	Supportive and transformational leadership	
Heponiemi T, Elovainio M, Pekkarinen L, Sinervo T, 2008 [70]	Journal of Community Psychology	Fair treatment and management protected against work interference with family when demands were low but were unable to buffer against the negative effects of high job demands.	Supportive and transformational leadership	
Al-Hussami M, 2009 [71]	Australian Journal of Advanced Nursing	Job satisfaction and perceived organizational support were most strongly related to nurses’ commitment to their organizations.	Supportive and transformational leadership	
Nielsen K, Munir F, 2009 [72]	Work & Stress	Followers’ self-ratings of self-efficacy mediated the relationship between transformational leadership style and positive affective well-being. Only limited evidence for a direct path between leadership behavior and positive affective well-being was found.	Supportive and transformational leadership	
Nielsen K, Yarker J, Randall R, Munir F, 2009 [73]	International Journal of Nursing Studies	Self-efficacy was found to fully mediate the relationship between transformational leadership and well-being and team efficacy was found to partially mediate the relationship between transformational leadership and job satisfaction and fully mediate the relationship between transformational leadership and well-being.	Supportive and transformational leadership	
Testad I, Mikkelsen A, Ballard C, Aarsland D, 2009 [74]	International Journal of Geriatric Psychiatry	QPS Nordic subscales significantly associated with stress in care staff were those associated with leadership.	Supportive and transformational leadership	
Winslow JH, Nielsen K, Borg V, 2009 [75]	Journal of Advanced Nursing	The more supervisors felt supported by fellow supervisors, the more their subordinates felt support by them.	Supportive and transformational leadership	

### Description of the articles

Most of the studies of this review were completed in North America (61%, n=36): in the USA 28 and in Canada 8. Thirty percent were completed in Europe (18 studies): Sweden (n=7), Denmark (n=5), Norway (n=3), Finland (n=2) and UK (n=1). One study was conducted in Australia, one in New Zealand, one in Egypt and one in Taiwan. English language as an inclusion criterion may be one reason of the large number of articles from North America.

The articles included in the review were published in 38 different scientific journals. This shows that publishing on elderly care management research has spread to many journals. Nursing was mentioned in title in 40% (n=23) of the journals and 24% (n=13) of titles referred to gerontological issues. The titles of 26% (n=15) of journals included the words management, administration/administrative, leadership, directors or economics. From this perspective institutional care management appears mainly as a question of nursing science.

Institutional care management research was quite rare in the early 2000s but increased in the following ten years. In the last years of the observation period especially the number of publications increased considerably. For instance, in the period 2000–2010, 65% of studies were published after 2005 and in the period 2006–2010, 54% of publications were published in 2009 and 2010.

The most common theoretical background of the studies in this review was a literature review of existing research (n=35, 60%). Nine of these articles included a description of some local or national program (e.g. a quality improvement program). One fifth of the studies (n=12, 21%) were based on a theoretical or practical model: Three studies were based on an older theoretical model by another researcher, and the rest were based on theoretical model constructed at least partly by the authors. Actual theories were identified in seven studies (13%). Complex adaptive system theory was used in five studies, action theory and social exchange theory in one study each. It can be said that research on institutional elderly care management is quite a new context in management research, and has mostly been based on earlier research or descriptions of practical reforms in long-term care but is developing in a more theoretical direction.

Questionnaires were used in 34% (n=20) of the studies and interview in 29% (n=17). Mixed methods were used in 31% (n=18) of the studies: registers in five, observation in three, interview in eleven, questionnaire in five and documents in six studies. Data was analyzed with statistical methods in 55% (n=32) of the studies. In 22% (n=13) of the studies data were analyzed using content analysis. Grounded theory was used in 7% (n=4) and ethnography in one study. Other methods included case studies, a modified phenomenographical approach and thematic analysis. It can be seen that during 2000–2010 institutional care management research has been based on very traditional research methods of collecting and analyzing empirical data. The background information on the studies is summarized in Table 3.

**Table 3:** Characteristics of studies of the systematic literature review

	**2000-2002**		**2003-2005**		**2006-2008**		**2009-2010**		**Total**	
	**n**	**%**	**n**	**%**	**n**	**%**	**n**	**%**	**n**	**%**
**Theoretical framework**										
Literature	4	100	8	50	11	62	12	60	35	60
Modified or self-made model			1	6	3	16	5	25	9	16
Theory			4	25	2	11	1	5	7	12
Theoretical model			1	6	2	11			3	5
Other			2	13			2	10	4	7
Total	4	100	16	100	18	100	20	100	58	100
**Data Collection**										
Questionnaire	2	50	4	25	5	28	10	50	21	36
Multi-method	1	25	7	44	8	44	2	10	18	31
Interview	1	25	4	25	4	22	7	35	16	27
Registers							1	5	1	2
Other			1	6	1	6			2	4
Total	4	100	16	100	18	100	20	100	58	100
**Data Analysis**										
Statistical	3	75	8	50	7	39	13	65	31	53
Content analysis			4	25	6	33	3	15	13	22
Grounded theory			1	6	1	6	2	10	4	7
Ethnography					1	6			1	2
Other	1	25	3	19	3	16	2	10	9	16
Total	4	100	16	100	18	100	20	100	58	100

## Results and discussion

The research on institutional elderly care management responded somewhat to the challenges mentioned in policy documents. However, some of the challenges were studied broadly and some were paid only minor attention. Further studies within the category of challenges rarely focused on the core items mentioned in the policy documents. Out of the 58 studies in this review 67% (n=39) included the issue of key challenges noted in international level long-term care policy documents. Concerning the challenges mentioned in policy documents quality management research was the most studied subject (29%, n=17). The second most common subject was workforce management (26%, n=15). Although integrated care was the most mentioned challenge in policy documents, integrated care management research (n=4) was very rare. Productivity received attention in two studies and ICT management in only one. Altogether, institutional care management research focused mainly on first-line management or charge nurse issues (93%, n=54). Only three studies addressed questions of strategic management. This may also be connected to the invisibility of productivity management research. In the following we will briefly present the main results of the studies, starting from the most studied to the least studied key challenges mentioned in policy documents. After that, the studies concerning challenges that were missing in policy documents will be shortly presented. Finally, the quality of this literature review will be discussed.

***In the quality management*** sub-category the need for systematic quality assurance was the most mentioned concern in long-term care policy documents. Only one of the studies in this review focused on this question. Kjos et al. [24] investigated first-line leader’s role in quality work. According to the results quality work was fragmented rather than comprehensive and systematic. First-line leaders reported that the municipalities delegated implementing national policies to the first-line leaders, but they received little support for their quality work from middle and top management.

The second sub-category in quality management mentioned in long-term care policy documents was the challenges in goal-setting. One article focused on goal setting management. Baier et al. [25] researched the effect of the level of ambition on the quality of resident’s outcome. The results indicated that nursing homes with ambitious targets demonstrated greater improvement than their peers with less ambitious targets. The results support the view of long-term care policy documents that goal-setting in quality work is important.

In addition, the need for valid quality measurement indicators was mentioned quite frequently in long-term care policy documents. Only one article focused on this theme. Parmelee et al. [26] researched clinical leaders’ relationship with a national quality measurement tool and reported that the relationship was complicated. Respondents valued the information it provided, but identified a number of weaknesses like validity, data accuracy and timeliness of assessments. These results strongly support the view of policy documents regarding the challenge in quality measurement and the need to develop the validity of the indicators. In many of the articles included in this literature review quality was measured with diverse quality indicators. These indicators mainly emphasized clinical practices and health indicators like Minimum Data Set (n=5) or assessed the outcomes of implementing evidence based health practices (n=2), clinical practices (n=3) or health indicators (n=3) at ward level. In terms of quality management, the expansion of the research to aspects other than health as part of well-being has not yet happened.

Besides those challenges mentioned in the policy documents, two other quality management issues were addressed in the studies included in this review: management factors influencing quality of resident outcomes (n=7) [27-33] and management factors associated with successful quality work implementation (n=7) [34-40]. According to the findings of these studies it seemed that for instance low turn-over of managers, their support for staff and good communication between managers and staff improved both the implementation of quality work and the quality of resident outcomes.

***The workforce management category*** included research on workforce training, stability and division of labor management. Management of updating training was researched in four studies. These studies explored the outcomes of a development program for managers and staff [42], reasons to increase staff attendance on training programs [44], staff’s capacity to identify their learning needs [41] and staff attitudes to education and supervision [43]. These results support the view of policy documents that updating training enhances the quality of care [42]. The results also show that the role and attitude of the manager are important for successful training interventions by staff [41,43,44].

The connection between leadership and workforce stability and accessibility was one of the most studied issues addressed in the challenges and emphasized in policy documents. The most common approach to stability was to study the reasons associated with staff turnover [45,46,48,49] and accessibility [47] and manager turnover [50-53]. For example, reward-based administrative climates [45] and consensus and supportive management [46,48] were found to be negatively associated with staff turnover. Opposite results were also reported, i.e. that there is no relationship between leadership practices and turnover intentions among staff [47].

Division of labor was mentioned in the policy documents as one of the ways to tackle workforce shortages in long-term care. This theme was addressed in two studies [54,55]. Both of these discussed delegation processes in care practices between charge nurse and unlicensed assisting personnel in the USA. Corrazzini et al. [55] researched the perceptions of registered nurses in leadership roles regarding barriers to and benefits of delegation. The results noted the benefits of delegation, because the staff to whom care was delegated gained a sense of empowerment [55]. Siegel et al. [54] were interested in the organizational, managerial and nurse-level factors associated with the nurse’s role as supervisor in a nursing home. The results showed that the nurses perceived their role as very extensive and complex but supportive management behavior can mediate role challenges to nurses struggling with role demands [54]. The results support the policy documents’ view of the benefits of the division of labor and the importance of management in this practice.

***Integrated care*** in long-term care was the most mentioned challenge in the policy documents. The biggest challenge articulated in the policy documents was lack of co-operation between professionals, organizations and families. Management of co-operation between these different actors was researched in two studies. Wilson [20] was interested in factors that may be significant in successful co-operation between nurses, residents and families in nursing homes and Miller [21] examined factors affecting successful partnerships between nursing home and hospice. The results demonstrated that leadership created the atmosphere in which relationships developed [20] and that similar visions of diverse actors and administrators demonstrated openness and support were associated with successful collaboration between nursing home and hospice [21].

Henriksen and colleagues in two articles [18,19] studied the understanding of managers and politicians about caring for older people. Elderly care services were regarded as a complex and fragmented organization lacking clear goals, structures and leadership. However, the managers and politicians believed these organizations were also willing to collaborate with each other [18,19]. Altogether, integrated care management research emphasized essential views like the importance of leadership in co-operation, the reality of fragmented organization and lack of common goals for care. These issues were also emphasized in policy documents. Despite extensive attention in policy documents integrated care management research was still quite scarce.

***Productivity management*** in long-term care was addressed in two studies, which is rare given the importance of long-term care productivity development noted in the policy documents. The study completed in New Zealand illuminated the facility managers’ opinion of financial sustainability in New Zealand [23]. Managers mentioned that the inadequate funding toward the growing costs of providing long-term care and occupancy levels is the most prominent management challenge in long-term care facilities. The second study conducted by Rantz et al. in the USA [22] handled the efficiency of nursing home care. This study described different factors, for example cost of care and leadership, with good, average and poor resident outcomes. According to the results leadership that is willing to embrace quality improvement, group processes and the quality of the basics of care was associated with good resident outcomes. The study does not directly demonstrate a connection between management and costs, but it does demonstrate that good care is not more expensive than bad care.

***ICT management*** was not addressed in any of the research reviewed. One study in Norway touched on this issue by investigating quality improvement activities of first-line leaders in long-term care [56]. This study, based on the Total Quality Management approach, showed that nursing homes made greater progress in implementing the general quality improvement components (e.g. priority of quality) than they did in implementing the technical quality improvement components that require training in tools and techniques. These results support the view of the policy papers that the use of ICT enhances the quality of management but that more extensive use requires training and available tools.

One-third (n=19) of the articles did not include the issue of the main challenges mentioned in policy documents. These studies can be divided into two categories: the first category included studies focusing on the leader’s own opinion of her/his skills, orientation, job satisfaction or role and style of management in the context of long-term care [57-64]. These articles described for instance different leadership styles or roles in managers’ work. The second category included human resource management studies which focused mainly on supportive or transformational leadership [65-75]. The articles mainly highlighted the need of supervisory support for staff in long-term-care settings. Quite often in these articles the support was connected to the wellbeing of nurses and their commitment to organization.

### Assessment of the quality of this review

The choice of policy documents and the categorizing of the challenges addressed in those documents depend on researchers’ interpretations. These interpretations can be criticized, although they were based on researchers’ scientific orientation in health and social management sciences. The actual review follows Petticrew’s [17] p.284-287 systematic literature protocol. The quality of this systematic literature review can be accessed using following criteria [76]: 1) the research question and inclusion criterion, 2) the search strategy, 3) the scientific quality of the data and 4) the analysis of the data.

The research question and inclusion criteria (empirical research of long-term care management, refereed article, published in English, published between 2000 and November 2010) were established prior to the review. After the search and selection process the list of studies included and excluded was compiled. If the appropriateness of an article for final data was uncertain, the researcher team strove for consensus.

The search strategy was implemented with a library information specialist. The search was conducted in two different electronic sources. The key words chosen are widely used in long-term care management research. In spite of a sensitive search strategy, it is possible that some important publications were overlooked and the search focused on the organization or ward level management issues and broader management orientation were not included. The search also showed that there is no national or international consensus of definitions of main key words such as terms referring to elderly or institutional care.

The quality of data was confirmed by including only refereed articles. No other quality analysis of the articles was made. The methods or analysis of studies was not a criterion for exclusion. Both qualitative and quantitative studies were included. The culture of USA in institutional elderly care context and research may be over-emphasized in the analysis of the systematic review because many of the studies were conducted there.

Mainly the analysis of data was unambiguous. In some case the studies were connected to more than one category of the framework (for example both quality work management and ICT management). In that case the main category was chosen by the main emphasis of the results of the article.

## Conclusions

The aim of the review was to compare institutional elderly care management research with the long-term care challenges currently addressed in international level long-term care policy documents. As a summary of the findings, it can be said that institutional elderly care is quite a new context in management research, but the amount of research has increased during the last ten years and has developed towards more theoretically-based research.

Judging from the scope of the journals in which the reviewed articles were published, institutional elderly care management appeared mainly as an issue related to nursing science: 41% of the articles in this review were published in journals focusing on nursing sciences. Only a quarter were published in journals focusing on management issues. To enhance the understanding of the challenges faced by institutional elderly care management, the research needs to stress a more multidisciplinary orientation and management sciences, and further research based on management theories will be needed.

By comparing institutional care management research to the topics of long-term care policy documents, the research focused on quality management and workforce stability management at the micro-level. Research on integrated care, productivity, ICT and division of labor have been rare and more are needed. In addition, comparisons in international, national or regional levels in institutional elderly care management or studies focusing on strategic management in institutional elderly care were non-existent: mostly, the research focused empirically on organizational or ward level issues in single organizations.

To some extent, the weak match between the research and the challenges of institutional elderly care also support the need for better co-operation between research, policy and practice. This co-operation is the only way how research can serve policy and practice to develop evidence-based decision-making and offer evidence-based information for the implementation of policy into practice. This is also partly a challenge of scientific literacy. Managers and policymakers’ scientific literacy probably needs to be enhanced, but at the same time in the scientific literature researchers should pay more attention to managers and policymakers as they do to readers using the research findings.

It is easy to agree with the researchers’ calls to enhance the relationship between managers and policymakers [4] and managers’ roles to be active in building permanent collaboration with research [5]. One current and significant example of such a collaboration and steering mechanism is The Future of Ageing Research in Europe: A Road Map [77]. This agenda for European ageing research was designed in co-operation between researchers, policymakers, practitioners, business people and elderly people, and it also sets out some important questions for the future of institutional elderly care management research.

## Competing interests

The authors declare that they have no competing interest.

## Authors’ contributions

KK made substantial contributions to the design of the review, searched for and reviewed the literature and wrote the first draft of the manuscript. SR and AH conceived of the review and helped with its design, interpretation of results and writing manuscript. They have given final approval of the version to be published.
